# Exploring the interplay of glucose metabolism, insulin resistance, and neurodegenerative pathologies: insights from streptozotocin and hypoglycaemic in vitro models

**DOI:** 10.1007/s00702-025-02891-6

**Published:** 2025-02-11

**Authors:** Edna Grünblatt, Cristine Marie Yde Ohki, G. Angelika Schmitt-Böhrer, Peter Riederer, Susanne Walitza

**Affiliations:** 1 Department of Child and Adolescent Psychiatry and Psychotherapy, Psychiatric University Hospital Zurich, University of Zurich, Zurich, Switzerland; 2 Neuroscience Center Zurich, University of Zurich and the ETH Zurich, Zurich, Switzerland; 3 Zurich Center for Integrative Human Physiology, University of Zurich, Zurich, Switzerland; 4 Department of Psychiatry, Psychosomatics, and Psychotherapy, Center of Mental Health, University of Würzburg, Würzburg, Germany; 5 Department and Research Unit of Psychiatry, Syddansk University, Odense, Denmark; 6 Head of Translational Molecular Psychiatry Chair of ECNP iPSC Platform for Neuropsychiatry Network, Department of Child and Adolescent Psychiatry and Psychotherapy, Psychiatric University Hospital Zurich (PUK), University of Zurich (UZH), Wagistrasse 12, Schlieren, CH-8952 Switzerland

**Keywords:** Streptozotocin, In vitro, Neuroblastoma, SH-SY5Y, N2A, PC12, HT22, Astrocytoma, Primary cells, SK-N-MC, iPSC, Alzheimer’s disease, Neuropsychiatric disorders, Hypoglycaemia, Brain insulin resistance

## Abstract

Neurodegenerative diseases raise public health concerns. Recent evidence indicates that Alzheimer’s disease (AD) sufferers will triple by 2050. The rising incidence of dementia diagnoses raises concerns about the socio-economical and emotional impact of this uncurable illness, which reduces quality of life through cognitive decline. Although genetic and environmental factors may contribute to its aetiology, neuropathological mechanisms underlying these disorders are still under investigation. One is brain insulin resistance (BIR), which has been associated with clinical cognitive dysfunction and linked to mitochondrial dysfunction, neurogenesis deficits, and cell death. Not limited to neurodegeneration, these phenotypes have been associated with other neuropsychiatric disorders. Streptozotocin (STZ), a diabetes-causing drug that targets pancreatic β-cells, may imitate BIR in suitable models. From patients’ neuroimaging to in vitro approaches, scientists have been striving to understand the pathophysiology of such disorders at the behavioural, molecular, and cellular levels. Although animal models are useful for studying insulin resistance’s systemic effects, in vitro phenotypic research represents an alternative to study molecular and cellular aspects. STZ and hypoglycaemia-like scenarios have been successful for studying neurodegenerative disorders in primary cell culture (e.g., neuroblastoma cells) and patient-specific neural cell lines derived from pluripotent stem cells (iPSCs). Intriguingly, STZ treatment or hypoglycaemia-like conditions in a dish were able to induce AD pathological characteristics such Aβ plaque deposition and Tau protein hyperphosphorylation. Such approaches have shown potential in understanding molecular and cellular implications of metabolic changes in neuropsychiatric disorders, according to this review. Furthermore, these models may help identify novel treatment targets.

## Introduction

Insulin, predominantly synthesised by pancreatic β-cells, is essential for brain metabolism and neuronal functions, including neuroplasticity, which is critical for cognition and emotional behaviour. In Alzheimer’s disease (AD), decreased brain insulin levels are associated with elevated biomarkers such as amyloid β (Aβ) plaques, but also with neuro-psychiatric symptoms such as mood disorders and cognitive impairment, suggesting a possible connection between insulin resistance and neurodegeneration (Rhea et al. 2024). AD is a progressive neurodegenerative disease marked by a gradual deterioration of cognitive function, memory impairment, and changes in behaviour, ultimately resulting in substantial impairment of daily activities. It is the most common kind of dementia among the elderly, with incidence projected to rise in the next two decades (Li et al. 2022; Lanctot et al. 2024). AD is characterised by an accumulation of amyloid plaques and neurofibrillary tangles, which disrupt with neuronal communication and trigger inflammatory responses that lead to neuronal death (Scheltens et al. 2021). Nevertheless, the etiopathology has to be elucidated. Epidemiological studies indicated that individuals with AD dementia exhibited a higher prevalence of diabetes compared to controls (Lanctot et al. 2024). The common type II diabetes mellitus (T2DM), which arises with ageing owing to inadequate insulin secretion from beta cells resulting in insulin resistance, is hypothesised to be associated with late-onset AD through numerous genetic and molecular associations (Lemche et al. 2024). Brain insulin resistance (BIR), unlike T2DM, can manifest without peripheral diabetes and is posited to be associated with AD pathogenesis (Rhea et al. 2024; Andrade et al. 2024) through mechanisms such as neuroinflammation and oxidative stress, which compromise neuronal function (for overview see (Kakoty et al. 2023). The scientific literature also refers to BIR as type 3 diabetes mellitus (T3DM), which is associated with brain-predominant diabetes that contributes to AD (de la Monte 2023; Kciuk et al. 2024; Peng et al. 2024).The aggregation of Aβ peptides in BIR induces inflammatory responses that exacerbate neuronal integrity and cognitive function deterioration (Choi et al. 2020). Nonetheless, BIR does not appear to be unique to AD, since epidemiological research indicates a correlation between diabetes and an elevated risk of Parkinson’s disease (PD), with insulin resistance significantly contributing to the degradation of dopaminergic neurons (Kakoty et al. 2023; Sian-Hulsmann et al. 2024). Mitochondrial dysfunction and α-synuclein aggregation are important characteristics of PD, with BIR playing a role in both pathological alterations (Kakoty et al. 2023).

Several models have been proposed to induce BIR, including dietary models such as high-fat diets (HFD), the knockout of the insulin receptor (INSR), or the usage of streptozotocin (STZ), all of which are able to produce Alzheimer’s-like phenotypes (for review see (Salkovic-Petrisic et al. 2022; Rhea et al. 2024). STZ is a glucosamine-nitrosourea chemical substance originating from a soil bacteria, first recognised as an antibiotic but currently utilised predominantly for diabetes induction in research (Martin-Carro et al. 2023). Peripheral intraperitoneal injection of STZ promoted pancreatic β-cell death, subsequently leading to type 1 or type 2 diabetes (Wan Chik et al. 2022). Conversely, the intracerebroventricular (icv) administration of STZ mitigates peripheral effects and promotes BIR (Salkovic-Petrisic et al. 2013, 2022). When administered icv, STZ generates abnormalities in the brain that closely mimic sporadic AD, including cognitive impairments, neuroinflammation, oxidative stress, mitochondrial dysfunction and modifications in neurotrophic factors (Salkovic-Petrisic et al. 2013, 2022; Wan Chik et al. 2022; Hoyer and Becker 1966; Lackovic and Salkovic 1990). This model indicates an important link between the effects of STZ and BIR, since the pathophysiology resembles that of T2DM, characterised by poor insulin signalling (González et al. 2022). The correlation between insulin resistance and AD’s pathology underscores the possibility that metabolic dysfunction may intensify neurodegenerative processes, indicating that modulating insulin signalling might provide therapeutic strategies for mitigating AD development (Kakoty et al. 2023; Salkovic-Petrisic et al. 2022). Consequently, STZ serves as a significant experimental paradigm for investigating the convergence of insulin resistance and Alzheimer’s-like phenotypes and pathologies and was recently found to induce a PD-like model when administered intrastriatally (Osmanovic Barilar et al. 2024). The utilisation of STZ in animal models to investigate the trajectories of neurodegeneration alongside behavioural traits has been widely discussed in a previous review (Salkovic-Petrisic et al. 2022). Moreover, the model has been utilised for therapeutic testing (Salkovic-Petrisic et al. 2013).

In accordance with the principles of the 3Rs (Replace, Reduce, and Refine) for animal models, in vitro models have been proposed to reduce or replace some animal usage (Eggel and Würbel 2021). Furthermore, in vitro methodologies provide some benefits over animal models for examining molecular and cellular changes during neurodevelopmental processes and across time. This review will summarise recent findings related to STZ and insulin resistance in in vitro models, along with their molecular and cellular phenotypes associated with neuropsychiatric conditions, including AD.

## In vitro models of BIR and AD using STZ

In vitro investigations utilising STZ provide significant insights into neuronal cell culture, especially concerning cytotoxicity and insulin signalling. STZ serves as a valuable in vitro model for AD by eliciting several molecular and metabolic alterations that replicate AD pathogenesis, as well as generally promoting insulin resistance. Insulin resistance induction, neurogenesis malfunction, mitochondrial dysfunction, excitatory/inhibitory imbalance, and changes in the processing of amyloid precursor proteins are some of the key factors. Nonetheless, although STZ serves as a useful tool for modelling specific facets of neurodegeneration, its effects might alter markedly depending on the differentiation state of neuronal cells, hence confounding the interpretation of insulin signalling activities in these scenarios. Subsequently, these aforementioned points will be addressed in detail and summarized in Table 1.

**Table 1:** STZ-induced cellular alterations of neuropathological mechanisms in in vitro or ex vivo studies up to this date

In vitro model	Dose	Main Findings	Reference
Immature cerebellar granule neurons from Wistar rats	2.5–5 mM	↓ Dose-dependent cell viability	Genrikhs et al. 2017
	3–4.5 mM	↑ Intracellular Ca^2+^	
	4.5 mM	↓ Mitochondrial membrane potential	
	3 & 4 mM	↓ Cell viability	Isaev et al. 2018
	4 mM	↑ Intracellular Ca^2+^	
Mouse N2A neuronal cells	50–1000 µM	↓ Cell viability ↑ Cytotoxicity	Biswas et al. 2016
	10–1000 µM	**↑ Phosphorylated Tau** ↓ Glucose uptake ↓ Mitochondrial fraction of cytochrome c ↑ DNA damage	
	50 & 100 µM	↑ Cytosolic fraction of cytochrome c	
	50–1000 µM	**↑ Amyloid aggregation** ↓ Levels of choline ↓ Mitochondrial membrane potential	
	100 & 1000 µM	**↑ Acetylcholinesterase activity** ↑ Apoptosis	
	15 µmol/L	↑ Apoptosis ↓ Mitochondrial membrane potential ↑ Mitochondrial ROS production ↓ ATP production ↓ Neurite outgrowth & neuronal differentiation	Chen et al. 2020
	0.01–5 mM	↑ Neurite outgrowth	Nishimoto et al. 2016
	10 mM	↑ Cytotoxicity and apoptosis	
	5 mM	↑ Phosphorylated Akt & GSK3β	
Hippocampal NSCs from Wistar rats	1–10 mM	↓ Number of neurospheres	Sun et al. 2018
	2.5 mM	↓ Diameter of neurospheres ↓ Cell proliferation ↓ Neuronal differentiation ↓ Gene and protein expression of GLUT3 ↓ Gene and protein expression of Insulin receptor	
Rat pheochromocytoma PC12 cells	0.8 mM	↓ Cell viability ↑ Pyroptosis markers	Li et al. 2020
HT22 neuronal cells	5 & 10 mM	↓ Cell viability	Park et al. 2020
	10 mM	↑ Apoptosis **↑ Phosphorylation of Tau epitopes** ↓ Synaptic markers (PSD-95) Altered mitochondrial morphology ↓ Intracellular ATP ↑ Intracellular ROS	
Primary rat hippocampal neurons	0.78–25 mM	↓ Cell viability	Bhuvanendran et al. 2022
	8 mM	**↑ Gene expression of MAPT**,** APP** & GSK3α/β **↑ Aβ-positive cells**	
Rat astrocytoma cells (C6)	100 µM	↓ Neurotrophic factors (BDNF & GDNF) Nitrostative stress: ↑ iNOS & nitrite Altered insulin signalling: ↓ IR, IDE and phosphorylated IRS-1, GSK3α/β & Akt **Altered amyloidogenic pathway: ↑ APP**,** Aβ1–42 & BACE-1** ↑ Intracellular ROS Astroglial activation: ↑ GFAP & COX-2 ↑ NF-kB translocation index ↑ TNF- α	Rajasekar et al. 2016
Primary astrocytes from Wistar rats	250 µM	Altered expression of astrocytic markers: ↑ GFAP; ↓ S100β Altered glutamate metabolism: ↑ glutamate uptake; ↓ activity of glutamine synthetase **Altered Aβ metabolism: ↑ Intracellular Aβ1–40 levels;** ↓ IDE & NEP	Taday et al. 2024
CA1 pyramidal neurons of rat hippocampal slices	1000 µM	↓ Amplitude of eEPSCs, AMPAR-mediated eEPSCs and eIPSCs ↑ eEPSC paired pulse ratio	Ju et al. 2016a
		↓ Frequency and amplitude of spontaneous excitatory postsynaptic currents (sEPSCs) & miniature excitatory postsynaptic currents (mEPSCs)	Ju et al. 2016b
CA1 pyramidal neurons of hippocampal slices from STZ diabetic mice	150 mg/kg	↑ Amplitude of action potentials & membrane potentials ↑ Frequency of sEPSCs	Fang et al. 2024
Human neuroblastoma cells (SK-N-MC)	2.5 & 5 mM	↑ Cytotoxicity	Plaschke and Kopitz 2015
	1 mM	↓ Intracellular ATP levels ↓ Phosphorylated GSK3β & ↑ total GSK3β **↓ Alpha-secretase activity in APP** **↑ Beta-secretase activity in APP** **↑ Aβ peptide (betaA4)**	
Human neuroblastoma cells (SH-SY5Y)	1–10 mM	↓ Cell viability	Bagamery et al. 2020; Bagamery et al. 2021
	3–10 mM	↑ Cytotoxicity	Bagamery et al. 2020
	1–2.5 mM	↓ Cell viability ↓ Cytotoxicity ↑ Intracellular ROS ↓ Mitochondrial membrane depolarization	Shandilya et al. 2021
	2.5 mM	↑ Endoplasmic reticulum stress ↑ Phosphorylated GSK3α/β	
	1 & 1.5 mM	**↑ Total intracellular Tau**	

AD pathology highlighted in **Bold**

### Cytotoxicity, apoptosis and neurogenesis effects of STZ

STZ exerts a pronounced cytotoxic impact on the survival of neuronal cells in culture, notably on immature neurons (Genrikhs et al. 2017; Isaev et al. 2018). Research indicates that STZ causes apoptosis and hinders neurogenesis, with effects differing according to neural maturity and cell type (Genrikhs et al. 2017; Isaev et al. 2018).

In primary immature cerebellar granule neurons derived from Wistar rats, STZ doses of 2–5 mM resulted in a dose-dependent reduction in cell viability (Genrikhs et al. 2017). Treatment with either 1mM pyruvate or 0.01mM insulin promoted partial protection against cell death (Genrikhs et al. 2017). STZ-induced neurotoxicity in vitro was found to be dependent upon the neuronal age (Isaev et al. 2018). In initial cultures of cerebella from Wistar rats, mature cerebellar granule neurons exhibited greater sensitivity to STZ-induced neurotoxicity compared to immature neurons (Isaev et al. 2018). In mouse N2A, rat pheochromocytoma PC12, and human neuroblastoma (SK-N-MC & SH-SY5Y) neuronal cells, STZ reduced cell viability and heightened oxidative stress and the activation of apoptotic pathways at relatively lower doses, likely due to the proliferative nature of these cells in contrast to differentiated neurons, which appear to be less impacted (Biswas et al. 2016; Plaschke and Kopitz 2015; Bagamery et al. 2020, 2021; Chen et al. 2020; Li et al. 2020; Shandilya et al. 2021). Like N2A and neuroblastoma cells, immortalised primary mouse hippocampal HT-22 neuronal cells exhibited dose- and time-dependent neurotoxicity to STZ (2.5–10 mM), along with apoptosis (Park et al. 2020). In N2A cells, STZ (0.01-5 mM) induced a dose-dependent enhancement of neurite outgrowth without affecting cell viability or lactate dehydrogenase (LDH) activity (Nishimoto et al. 2016). This research showed that STZ promotes cell growth instead of inducing cell death. Nonetheless, treatment of the cells with 10mM STZ resulted in increased cell death accompanied by elevated LDH release (Nishimoto et al. 2016).

Furthermore, STZ administration to isolated adult mouse hippocampal neural stem cells (NSCs) led to reduced proliferation and differentiation, predominantly impacting the production of new neurons (Sun et al. 2018). Specifically, STZ treatment diminishes the proliferation of adult hippocampal NSCs, as indicated by a reduction in both the size and quantity of neurospheres (Sun et al. 2018). It predominantly influences the formation of new neurons while preserving astrocyte differentiation (Sun et al. 2018). This indicates the impact of STZ on adult neurogenesis at different stages of development.

STZ’s neurotoxic effects accentuate its involvement in neuronal cell death and possible ramifications for neurodegenerative diseases (Goel et al. 2022; Wan Chik et al. 2022). Nonetheless, it is essential to consider that these neurotoxic effects were dose-dependent and determined by the cellular state (proliferation, differentiation, or maturity). Conversely, although STZ is recognised for its neurotoxic effects, some research indicate that certain therapies may alleviate these effects, suggesting a possibility for therapeutic interventions in STZ-induced neurotoxicity. In the N2A model, isoquercetin, a flavonoid, had protective effects against STZ-induced cytotoxicity and apoptosis (Chen et al. 2020). While STZ reduced the differentiation and neurite outgrowth of N2A cells, isoquercetin administration mitigated the effects of STZ (Chen et al. 2020). In SH-SY5Y cells, vitamin K2 mitigated the cytotoxicity-induced cell death produced by STZ (Shandilya et al. 2021).

### Mitochondrial dysfunction and oxidative stress induced by STZ treatment

Mitochondrial dysfunction and oxidative stress are some processes that have been associated to the pathogenesis of AD and other neuropsychiatric disorders (Pinto Payares et al. 2024; Papageorgiou and Filiou 2024) and scientifically explored by using in vitro models and STZ (Dewanjee et al. 2022; Moreira et al. 2007). As an example, Genrikhs and colleagues have hypothesised that STZ-induced mitochondrial dysfunction may result in calcium (Ca^2+^) excess in cerebellar granule neurons. In this study, although cell death was not detected after a 5-hour STZ exposure of primary cerebellar neuronal cells, an increase in Fluo-4-labelled intracellular Ca^2+^ ions was found alongside a concurrent drop in tetramethylrhodamine ethyl ester (TMRE) signals, which indicate mitochondrial membrane potential (Genrikhs et al. 2017). The elevation of Ca^2+^ levels in immature neurons may cause damage to mitochondria and other organelles, as previously demonstrated (Genrikhs et al. 2017).

Moreover, in another report, STZ-induced mitochondrial impairment and modified insulin signalling pathways facilitated neurodegenerative alterations, which associates STZ exposure with AD-like pathology (Park et al. 2020). Specifically, STZ-treated HT-22 cells exhibited Drp1-mediated mitochondrial fragmentation, which was associated with reduced intracellular ATP levels and elevated intracellular reactive oxygen species (ROS) levels (Park et al. 2020). Similarly, in SK-N-MC cells, STZ induced a significant decrease in ATP levels, indicating mitochondrial dysfunction (Plaschke and Kopitz 2015).

Comparable mitochondrial stress and translocation of cytochrome c into the cytosol were seen in N2A cells treated with STZ (Biswas et al. 2016). Chen et al. demonstrated in N2A cell culture that STZ treatment resulted in elevated ROS, which may be mitigated by isoquercetin treatment, in parallel to its aforementioned beneficial effects in N2A differentiation and neurite outgrowth (Chen et al. 2020). In SH-SY5Y cells, STZ elicited ROS production and mitochondrial depolarisation, which was mitigated by vitamin K2 therapy (Shandilya et al. 2021). Consequently, it may be concluded that STZ may alter mitochondrial function, resulting in elevated oxidative stress and the production of ROS.

### Electrophysiological changes after STZ treatment

In immature cerebellar granule neurons, STZ treatment led to a substantial rise in Fluo-4, indicating a shift in Ca^2+^ influx and neuronal activity, but in mature cells, only a slight increase was noticed (Isaev et al. 2018). The impact of STZ on electrophysiological activity was assessed in hippocampus slices from both male and female Wistar rats using the whole cell patch clamp method. STZ hinders synaptic transmission in rat hippocampal neuron slices, as seen by evoked excitatory postsynaptic currents (eEPSCs) and evoked inhibitory postsynaptic currents (eIPSCs) in CA1 pyramidal neurons, influencing both excitatory and inhibitory pathways (Ju et al. 2016a). This inhibition indicated that STZ impacted the projections of glutamatergic and GABAergic neurons (Ju et al. 2016b). Similar results were seen in hippocampus slices derived from STZ diabetic mice (Fang et al. 2024). No substantial alterations in electrophysiological parameters were detected during the initial week following hyperglycaemia; nevertheless, considerable elevations in membrane potentials and action potentials were documented in the subsequent week (Fang et al. 2024). CA1 neurons exhibited elevated EPSCs in the second week following the onset of hyperglycaemia. The patterns of neural activity in the aforementioned experiments resemble those reported in AD (Selkoe 2019; Zott et al. 2019).

### Overlap of AD pathology with STZ effects

Given the unique AD neuropathology characterised by the production of Aβ plaques and hyperphosphorylation of Tau proteins, these markers and others have been examined in STZ in vitro models (see highlighted in Table 1). In N2A cells, STZ caused a notable decrease in choline levels, an elevation in acetylcholinesterase (AchE) activity, Tau phosphorylation, and amyloid aggregation (Biswas et al. 2016). Although the AchE inhibitor donepezil effectively mitigated STZ neurotoxicity, choline levels, AchE activity, and mitochondrial stress, apoptosis, as shown by caspase-3 expression, remained un-improved (Biswas et al. 2016). STZ in neuroblastoma SK-N-MC cells markedly elevates beta-secretase (BACE-1) activity and Aβ levels, while reducing alpha-secretase activity, hence facilitating amyloidogenic processing (Plaschke and Kopitz 2015). In SH-SY5Y neuroblastoma cells, STZ induced overall Tau accumulation and a modest rise in Aβ42 concentrations, while vitamin K2 mitigated these changes (Shandilya et al. 2021). Lastly, in the hippocampus cell lines HT-22, STZ-induced synapse loss and aberrant tau hyperphosphorylation were observed (Park et al. 2020). In line with in vitro findings, icv STZ-animal models also showed changes in AD-related proteins, such as elevated Aβ and phosphorylated Tau (Ravelli et al. 2017; Salkovic-Petrisic et al. 2013, 2022).

Furthermore, glial cell dysfunction has been pointed out as potential players in the development of AD (Uddin and Lim 2022; Litwiniuk et al. 2023). For instance, in primary rat astrocytes, STZ at 100 µM significantly reduced the gene and protein expression of the neurotrophic factors BDNF and GDNF (Rajasekar et al. 2016). Besides, the protein expression of insulin receptor (IR), phosphorylated insulin receptor substrate-1 (pIRS-1), phosphorylated GSK3α/ β and insulin-degrading enzyme (IDE) were also downregulated in these STZ-treated astrocytes, as opposed to amyloid precursor protein (APP), Aβ1–42, BACE-1, suggesting dysregulations in insulin and amyloidogenic pathways (Rajasekar et al. 2016). In the same study, some results indicate STZ-induced oxidative stress and inflammatory response in astroglial cells, such as increased intracellular ROS levels, mRNA expression of inducible nitric oxide synthase (iNOS), nitrite release, NF-kB translocation and TNF-α, GFAP and COX-2 expression (Rajasekar et al. 2016).

Moreover, STZ has been found to affect astrocytic functionality by enhancing glutamate uptake in STZ-treated astrocytes and led to decreasing levels of neprilysin (NEP) and IDE associated to augmented intracellular content of Aβ1–40 (Taday et al. 2024). These results align with previous findings of STZ-induced gene expression of APP, GSK3α, GSK3β and microtubule-associated protein Tau (MAPT) in primary hippocampal neurons (Bhuvanendran et al. 2022) and with the aforementioned findings from Rajasekar et al. (Rajasekar et al. 2016). These led the authors to hypothesize that these STZ-induced alterations in astrocytes could play a role in the AD pathogenesis (Bhuvanendran et al. 2022).

### Insulin resistance and STZ

Based on the hypothesis that AD pathogenesis arises from BIR and modelling this in animals by the administration of low doses of STZ, several research have been undertaken to examine it in vitro. The application of STZ to N2A cells resulted in reduced glucose uptake (Biswas et al. 2016). In differentiated SH-SY5Y cells treated with STZ, a rightward shift in the dose-response curve of insulin-induced GSK-3 phosphorylation was observed, which suggests the beginning of insulin resistance (Bagamery et al. 2021). In isolated adult hippocampus NCSs of mice, STZ caused a considerable decrease in the mRNA and protein expression of the glucose transporter (GLUT3), which was similarly observed in differentiating cells (Sun et al. 2018). Nonetheless, markedly reduced insulin receptor expression was exclusively observed in the NSCs, rather than in the differentiating cells (Sun et al. 2018).

Based on the aforementioned findings in vitro, several elements observed in vivo in STZ animal models and human AD patients appear to be replicated using neuronal cell cultures treated with STZ. Nevertheless, although STZ effectively models sporadic AD, it is crucial to account for the heterogeneity in responses across various experimental conditions, as this may affect the interpretation of findings and therapeutic consequences.

## In vitro models of BIR and AD with low glucose and hypoglycaemia induction

Investigating glucose levels in vitro is essential for understanding their impact on neuropsychiatric disorders. Both hyperglycaemia and hypoglycaemia have been linked to various mental health issues, including dementia, depression and anxiety, highlighting the importance of maintaining balanced glucose levels for optimal neuronal function (Genis-Mendoza et al. 2022; Xie et al. 2022; Huang et al. 2022). Elevated glucose levels can disrupt neuronal activity, potentially leading to cognitive decline and mood disorders, while low glucose levels may manifest as confusion and irritability, often misinterpreted as psychiatric symptoms (Yonamine et al. 2023; Orquin and Kurzban 2016). Moreover, insulin resistance, which impairs glucose uptake in the brain, is associated with cognitive decline and mood disorders, emphasizing the need for metabolic health in mental well-being (Genis-Mendoza et al. 2022; Xie et al. 2022; Huang et al. 2022). Episodes of hypoglycaemia, particularly in individuals with diabetes, has been shown to contribute to cognitive impairments, further underscoring the relationship between glucose metabolism and neuropsychiatric conditions (Huang et al. 2022; Ye et al. 2024). Therefore, in vitro studies focusing on glucose levels can provide valuable insights into the mechanisms underlying these disorders, potentially revealing therapeutic targets for intervention and management. This section will provide an overview of the research linking altered glucose levels in vitro to potential neurodegeneration and AD-like pathology (summarized in Table 2).

**Table 2:** Cellular neuropathological alterations of hypoglycaemia-like conditions in cell culture

In vitro model	Cell culture medium	Main Findings	Reference
Rat pheochromocytoma PC12 cells	Glucose-free medium	↓ Cell viability ↑ LDH leakage Altered Wnt signalling: ↓ Gene and protein expression of Wnt3A & β-catenin ↑ Apoptosis	Xu et al. 2016
		↑ Apoptosis ↑ Necrosis ↓ Mitochondrial membrane potential ↓ ATP production ↑ Intracellular ROS	Liu et al. 2003
		↓ Cell viability ↓ Cell proliferation Altered apoptotic pathway: ↑ FOXO3-positive cells, ↓ Phosphorylated Akt & Bcl-2, ↑ Cleaved Caspase-3	Han et al. 2016
		↓ Cell viability ↑ Apoptosis ↓ Gene and protein expression of Bcl-2 ↑ Gene expression and activity of Caspase-3 ↓ Mitochondrial membrane potential ↑ Intracellular ROS	Yu et al. 2008
Mouse N2A neuronal cells	Glucose-free medium	↓ Cell viability ↑ Apoptosis ↑ Intracellular ROS ↓ ATP, ADP & ATP/ADP **↓ Monomeric Aβ** ↓ Autophagic flux	Chiang et al. 2023
	Glucose/pyruvate-deficiency media	↓ Mitochondrial membrane potential ↑ GSK3α/β & AMPK activity **↑ Phosphorylated Tau**	Lee et al.
Mouse N2A/APP695	Glucose-free medium	↓ Cell proliferation ↑ Apoptosis **↓ BACE-1 expression** Altered autophagy: ↑ LC3 & Beclin1	Chen et al. 2015
Human neuroblastoma cells (SH-SY5Y)	Glucose 0–1 mM	↓ Cell viability	Kobayashi et al. 2007
	Glucose 0 & 0.25 mM	↑ Apoptosis	
	Glucose 0-2.5 mM	↓ Mitochondrial activity	
	Glucose 2.5 mM	Gene expression dysregulation	
	Glucose 0.25–5.5 mM	↓ Cell viability ↑ Apoptosis ↑ Lactate levels	Nagy et al. 2019
Cortical human iPSC-derived neurons	Glucose 0.5-2 mM	↑ Neuronal degeneration	Huang et al. 2023
	Glucose 2 mM	↓ Synaptic density ↓ Neurite coverage Altered electrophysiology: ↑ Mean firing rate, ↓ synchrony index ↓ Mitochondrial membrane potential ↑ Oxidative stress ↓ O-GlcNAcylation **↑ Neurofibrillary tangles**,** phosphorylated Tau & Aβ** Mitochondrial O-GlcNAcylation-related dysfunction	

AD pathology highlighted in **Bold**

### Cytotoxicity, apoptosis and neurogenesis effects

PC12 cells cultivated in glucose-free medium, mimicking hypoglycaemia, exhibited a significant decrease in cell viability and an elevated production of LDH compared to control cells (Xu et al. 2016). Notably, the same study pointed out to the participation of the Wnt signalling in this context. More specifically, its activation by lithium chloride (LiCl), a Wnt agonist, counteracted those effects, whereas the application of DKK1, a Wnt antagonist, with LiCl attenuated this restorative effect (Xu et al. 2016). This was corroborated by reduced levels of the Wnt ligand and activator Wnt3A and β-catenin mRNA and proteins following culture in glucose-free media, while LiCl reversed this impact. Apoptosis was also found in PC12 cells following hypoglycaemia, which was mitigated by LiCl therapy (Xu et al. 2016). Similarly, Liu et al. observed that glucose deprivation in PC12 cells for 24 h resulted in both necrosis and apoptosis, possibly attributable to the build-up of ROS and depletion of ATP (Liu et al. 2003).

Culture of SH-SY5Y cells in low glucose (0–1 mM) for 24 h resulted in a substantial decrease in cell viability, correlating with the initiation of cell death and apoptosis (Kobayashi et al. 2007). Similarly, the creation of a hypoglycaemic environment in SH-SY5Y cells by the use of glucose-free medium, with glucose concentrations adjusted between 0.25 and 5.5 mM, has led to reduced cell viability, heightened apoptosis upon glucose consumption, and an increase in lactate levels (personal communication with Tamas Nagy, University of Pécs, Hungary (Nagy et al. 2019). Similar to PC12 and SH-SY5Y cells, N2A cells subjected to 24 h of glucose deprivation exhibited increased cell death and apoptosis (Chiang et al. 2023). N2A/amyloid precursor protein 695 (APP695) cells were subjected to oxygen-glucose deprivation to model cerebral ischaemia, resulting in a significant decrease in cell proliferation and an increase in apoptosis (Chen et al. 2015).

Notably, repeated glucose deprivation/reperfusion in PC12 cells resulted in greater cell mortality than glucose deprivation alone (Han et al. 2016). The forkhead box O 3 (FOXO3) transcription factor, involved in the regulation of cell apoptosis and survival, was observed to localise in the nuclei of cells subjected to glucose deprivation/reperfusion, coinciding with increased apoptosis (Han et al. 2016). This suggests that hypoglycaemia activates FOXO3, resulting in apoptosis.

However, the alleviation of glucose deprivation effects was achievable by researchers of this field using certain compounds. For instance, Salidroside, a phenol glycoside extracted from the root of *Rhodiola rosea*, utilised in Tibetan medicine, exhibits properties including anti-inflammatory, anti-hypoxia, and anti-oxidative effects, and may enhance serotonin levels with nicotinic cholinergic effects in the Central Nervous System (CNS) (Yu et al. 2008). It was observed that salidroside mitigates cell death and apoptosis following 24 h of glucose and serum deprivation in PC12 cells (Yu et al. 2008).

### Mitochondrial dysfunction and oxidative stress

Glucose-deprived PC12 cells exhibited an elevation in ROS levels and a decline in mitochondrial performance, whereas intracellular ATP levels rose during the initial 3 h, followed by a gradual decrease (Liu et al. 2003). Furthermore, the mitochondrial transmembrane potential (ΔΨm) decreased after 6 h (Liu et al. 2003). Concurrently to the previously stated effects of Salidroside on cell death and apoptosis during 24 h of glucose and serum deprivation in PC12 cells, pre-treatment averted changes in mitochondrial membrane potential and ROS generation in these cells (Yu et al. 2008). Similarly, glucose deprivation (0-2.5 mM) during 24 h in SH-SY5Y cells resulted in a dose-dependent reduction of 30% to almost 90% (Kobayashi et al. 2007). N2A cells undergoing 24 h of glucose deprivation exhibited a reduction in ATP levels accompanied with an increase in ROS (Chiang et al. 2023). Differentiated N2A neuroblastoma cells were cultivated in glucose/pyruvate-deficient medium in order to examine the potential link between hypoglycaemia/hypometabolism and the development of AD. Subsequent to the cultivation of N2A cells in glucose/pyruvate-deficient medium, the mitochondrial membrane potential decreased, concurrently with the activation of AMP-activated protein kinase (AMPK), an energy sensor (Lee et al. 2013). This indicates that hypoglycaemia causes energy stress in the cells. In cortical neurons generated from human induced pluripotent stem cells (iPSCs), low glucose concentrations led to mitochondrial abnormalities, as seen by mitochondrial membrane potential, oxidative stress induction, and Seahorse mitochondrial stress test measurements (Huang et al. 2023).

### Electrophysiological changes after glucose levels alterations

In cortical neurons derived from human iPSCs, low glucose concentrations resulted in a reduction of neurite length accompanied by partial fragmentation of neurites (Huang et al. 2023). Furthermore, the assessment of neuronal activity using multi-electrode arrays (MEA) revealed a decline in neuronal activity after 2 days of 2 mM low glucose treatment, followed by a progressive upsurge in activity that culminated in a 2.5-fold elevation in mean firing rate after 5 days (Huang et al. 2023). This change in mean firing may signify neuronal hyperexcitability, a characteristic of neurodegeneration shown in several in vitro and in vivo AD models (Huang et al. 2023). Furthermore, the synchronisation, indicative of synaptic function, was diminished in these cells due to the low glucose treatment (Huang et al. 2023). Therefore, these findings suggest that hypoglycaemia may compromise neuronal structure and function in human iPSC-derived cortical neurons.

### AD pathology overlaps to hypoglycaemia-like scenarios in vitro

In a recent study, N2A neuroblastoma cells that underwent 24 h of glucose deprivation exhibited impaired autophagic flux, resulting in decreased basal Aβ levels, mainly monomeric Aβ (Chiang et al. 2023). Differentiated N2A cells grown in glucose/pyruvate-deficient media exhibited elevated levels of tau phosphorylation at Ser262 and Ser396 (Lee et al. 2013). In the N2A/APP695 cells subjected to oxygen-glucose deprivation, BACE-1 levels were markedly reduced, while autophagy was elevated, perhaps resulting in neurodegeneration and increase Aβ accumulation (Chen et al. 2015). Reducing the glucose concentration to a crucial threshold (2 mM) elicited AD-like pathology in cortical human iPSC-derived neurons, but not in cerebellar neurons (Huang et al. 2023). Specifically, neurofibrillary tangles (NFT) and Tau AT8, including phosphorylated Tau at T181 and T231, were elevated, along with higher levels of both Aβ1–40 and Aβ1–42 (Huang et al. 2023). Refer to the summary highlighted in Table 2.

## Discussion

The present review highlights the essential function of glucose metabolism and insulin resistance in the pathogenesis of neurodegenerative disorders, including AD (see Table 3). The use of STZ and hypoglycaemic conditions in vitro has yielded significant insights into the cellular and molecular alterations that characterise these disorders. This review demonstrates that STZ not only simulates BIR but also triggers AD-like clinical characteristics, including Aβ plaque accumulation and tau hyperphosphorylation, in several neuronal cell cultures. Moreover, the effects of glucose restriction on neuronal survival, oxidative stress, and mitochondrial dysfunction underscore the need of preserving metabolic health for optimal cognitive performance. These in vitro models provide a viable framework for studying the causes of neuropsychiatric disorders and for identifying prospective treatment targets. These findings have significant ramifications, providing a means to create therapies that might influence glucose metabolism and insulin signalling, perhaps reducing the advancement of neurodegenerative disorders.

**Table 3:** STZ and hypoglycaemia in vitro models compared to AD-neuropathology findings

AD neuropathology	Finding in AD patients (reference)	STZ in vitro model (reference)	Hypoglycaemia in vitro model (reference)
Aβ accumulation	↑ (Scheltens et al. 2021)	↑ (Biswas et al. 2016; Bhuvanendran et al. 2022; Taday et al. 2024; Plaschke and Kopitz 2015)	↓ Monomeric Aβ (Chiang et al. 2023) ↑ (Huang et al. 2023)
Hyperphosphorylation of Tau	↑ (Scheltens et al. 2021)	↑ (Biswas et al. 2016; Park et al. 2020; Bhuvanendran et al. 2022; Shandilya et al. 2021)	↑ (Lee et al. 2013; Huang et al. 2023)
BACE-1	↑ platelet (Fu et al. 2023) ↓ postmortem frontal cortex (Decourt et al. 2013)	↑ (Rajasekar et al. 2016; Plaschke and Kopitz 2015)	↓ expression (Chen et al. 2015)
GFAP	↑ in blood (Holper et al. 2024)	↑ (Rajasekar et al. 2016; Taday et al. 2024)	n.d.
Cell death (Apoptosis, necrosis, autophagy)	↑ (Goel et al. 2022) miRNAs enriched in apoptosis pathways (Yoon et al. 2022)	(Genrikhs et al. 2017; Biswas et al. 2016; Isaev et al. 2018; Chen et al. 2020; Nishimoto et al. 2016; Li et al. 2020; Park et al. 2020; Bhuvanendran et al. 2022; Plaschke and Kopitz 2015; Bagamery et al. 2020; Shandilya et al. 2021)	(Xu et al. 2016; Liu et al. 2003; Han et al. 2016; Yu et al. 2008; Chiang et al. 2023; Chen et al. 2015; Kobayashi et al. 2007; Nagy et al. 2019; Huang et al. 2023)
Mitochondria dysfunction & oxidative stress	↑ Malondialdehyde, Superoxide dismutase (Zabel et al. 2018) ↓ cytochrome c oxidase (Morais et al. 2021)	(Genrikhs et al. 2017; Chen et al. 2020; Park et al. 2020; Rajasekar et al. 2016; Plaschke and Kopitz 2015; Shandilya et al. 2021)	(Liu et al. 2003; Yu et al. 2008; Chiang et al. 2023; Lee et al. 2013; Kobayashi et al. 2007; Huang et al. 2023)
Insulin resistance / hypoglycaemia	(Rhea et al. 2024; Peng et al. 2024; Lemche et al. 2024; Kciuk et al. 2024; Andrade et al. 2024; de la Monte 2023)	(Biswas et al. 2016; Sun et al. 2018; Rajasekar et al. 2016)	Generated by the model
GLUT3	↓ postmortem hippocampus & cortex (Kyrtata et al. 2021)	↓ hippocampal NSCs from Wistar rats (Sun et al. 2018)	n.d.

Abbreviations: n.d. no data available

While the review presents a compelling case for the use of STZ and hypoglycaemia induction in vitro to study neuropsychiatric disorders, there are significant concerns regarding the relevance and applicability of these models to human conditions. Neurodegenerative diseases such as AD are complex given their multifactorial conditions, which are affected by several genetic, environmental, and lifestyle variables. Relying heavily on STZ as a model may oversimplify the intricate pathophysiology of AD and other neuropsychiatric conditions. Furthermore, classical pharmacology’s dose- and time-dependence may not necessarily be applicable to in vitro models; hence, caution is warranted.

On the other hand, although the in vitro models discussed are useful for certain aspects of research, they often fail to replicate the complex cellular environments present in vivo. For instance, the absence of systemic interactions, such as those involving immune cells and other brain cell types, can limit our understanding of how neuroinflammation and other processes contribute to neurodegeneration. Furthermore, while most studies focus on the investigation of neuronal cell lines, glial cells are equally important for the homeostasis of the central nervous system and participate in the development of neuropsychiatric conditions (e.g., dementia), as reported in AD patients (Nordengen et al. 2019) and previously reviewed (Litwiniuk et al. 2023; Rodriguez et al. 2009; Uddin and Lim 2022). As an example, according to Kim et al., astrocytes and microglia may play both protective and toxic roles in AD models by regulating synaptic function, neuroinflammation and the deposition or degradation of Aβ plaques (Kim et al. 2018). In vivo studies showed STZ-induced glial dysfunction and activation (Wong et al. 2011; Luo et al. 2002; Hanani et al. 2014). However, the scientific community could benefit from more glia-specific in vitro investigations to better elucidate what are the direct effects caused by hypoglycaemia or STZ in astrocytes, microglia and oligodendrocytes, and how they can be detrimental or beneficial to their interaction with neurons.

Nevertheless, the cellular responses observed in isolated cultures may not translate to the holistic responses seen in a living organism, potentially leading to erroneous conclusions about the efficacy of therapeutic interventions. Expanding research to include more sophisticated models that incorporate these systemic interactions is crucial for bridging the gap between in vitro findings and in vivo realities, ultimately enhancing our understanding of neurodegenerative diseases. For example, personalized human iPSC-derived neural models can provide insights into patient-specific responses and disease mechanisms, allowing researchers to explore how genetic variations influence the progression of neurodegeneration and the effectiveness of targeted therapies. By utilizing these advanced models, scientists can better identify biomarkers for early detection and tailor treatments that are more effective based on individual patient profiles, paving the way for precision medicine in neurodegenerative disorders. Consequently, subsequent research necessitates the utilisation of advanced technology, including 2D and gradually complex 3D models employing personalised human iPSCs.

Despite certain limitations, this review shows that in vitro models can be a valuable pre-clinical tool for addressing the alarming rise in neuropsychiatric and neurodegenerative disorders in older adults, particularly when combining multiple perspectives to enhance disease prevention, early detection, and early intervention.
